# Author Correction: Open-3DSIM: an open-source three-dimensional structured illumination microscopy reconstruction platform

**DOI:** 10.1038/s41592-023-01996-8

**Published:** 2023-08-24

**Authors:** Ruijie Cao, Yaning Li, Xin Chen, Xichuan Ge, Meiqi Li, Meiling Guan, Yiwei Hou, Yunzhe Fu, Xinzhu Xu, Christophe Leterrier, Shan Jiang, Baoxiang Gao, Peng Xi

**Affiliations:** 1 Department of Biomedical Engineering, College of Future Technology, Peking University, Beijing, China; 2 National Biomedical Imaging Center, Peking University, Beijing, China; 3 Key Laboratory of Analytical Science and Technology of Hebei Province, College of Chemistry and Environment Science, Hebei University, Baoding, China; 4 Aix-Marseille Université, CNRS, INP UMR7051, NeuroCyto, Marseille, France; 5 Institute of Biomedical Engineering, Beijing Institute of Collaborative Innovation, Beijing, China

**Keywords:** Fluorescence imaging, Technology

Correction to: *Nature Methods* 10.1038/s41592-023-01958-0. Published online 20 July 2023.

Following publication of this article, Christophe Leterrier (Aix-Marseille Université, CNRS, INP UMR7051, NeuroCyto, Marseille, France) was added as an author of the paper for his contribution of Figure 2e. The author list, affiliations, acknowledgements and author contributions and have been updated accordingly.

In the originally published version of the paper, the caption for Figure 2e stated “The multicolor reconstruction of Open-3DSIM of the nuclear pore complex, actin filament and tubulin, excited at 488, 561 and 683 nm in wavelength, respectively.” This was incorrect as clathrin rather than nuclear pore complexes were labeled. This has been corrected to read: “The multicolor reconstruction by Open-3DSIM of a COS cell labeled for actin filaments (yellow), clathrin (cyan), and tubulin (red), excited at 488, 561 and 640 nm in wavelength, respectively.” Additional changes have been made to the “Sample preparation” subsection of Methods to clarify acquisition of Figure 2e.

In addition, in the original version of the main text, the authors stated “Next, according to the estimated frequency vector in the *xoy* and *yoz* plane, we designed a two-step filter in the frequency domain based on the notchfilter (Notch), apodization function (Apo), OTF and OTFnotch.” In the corrected version, they clarify that this was inspired by existing work. The text has been updated to read: “Next, inspired by Hifi-SIM^7^, we designed a two-step filter in the frequency domain based on the notch function (Notch), apodization function (Apo), optical conversion function (OTF) and OTF_notch_ according to the estimated frequency vector in the *xoy* and *yoz* plane.” All changes have been made in the HTML and PDF versions of the article.

