# Supplementary material for: Open-3DSIM: an open-source three-dimensional structured illumination microscopy reconstruction platform
Source: Nat Methods. 2023 Jul 20;20(8):1183–6. doi: 10.1038/s41592-023-01958-0 (PMC10406603; doi:10.1038/s41592-023-01958-0)
Supplement: Supplementary file 1 — Supplementary Fig. 1, Notes 1–11 and Tables 1 and 2. [file 41592_2023_1958_MOESM1_ESM.pdf]

nature methods

Brief Communication

https://doi.org/10.1038/s41592-023-01958-0

Open-3DSIM: an open-source three-dimensional structured illumination microscopy reconstruction platform

In the format provided by the authors and unedited

Supplementary Note

Supplementary Figure 1. Principle of 3DSIM 2
Supplementary Note 1. Principle of Open-3DSIM 3
Supplementary Note 2. Algorithm flow 8
Supplementary Note 3. The effect of spectrum optimization 9
Supplementary Note 4. Guide for choosing parameters of spectrum optimization 10
Supplementary Note 5. Comparison between single-layer and multi-layer SIM reconstruction 12
Supplementary Note 6. Simulation on Open-3DSIM 13
Supplementary Note 7. Comparison of Weiner-based 3DSIM algorithms 14
Supplementary Note 8. Comparison of various 3DSIM algorithms on Argolight 15
Supplementary Note 9. Comparison of various 3DSIM algorithms on various samples 16
Supplementary Note 10. Multi-color imaging and reconstruction 17
Supplementary Note 11. Dipole orientation imaging 18
Supplementary Table 1. Parameters and data origin used in our work. 19
Supplementary Table 2. List of the open-source data. 20

Supplementary Figure 1. Principle of 3DSIM

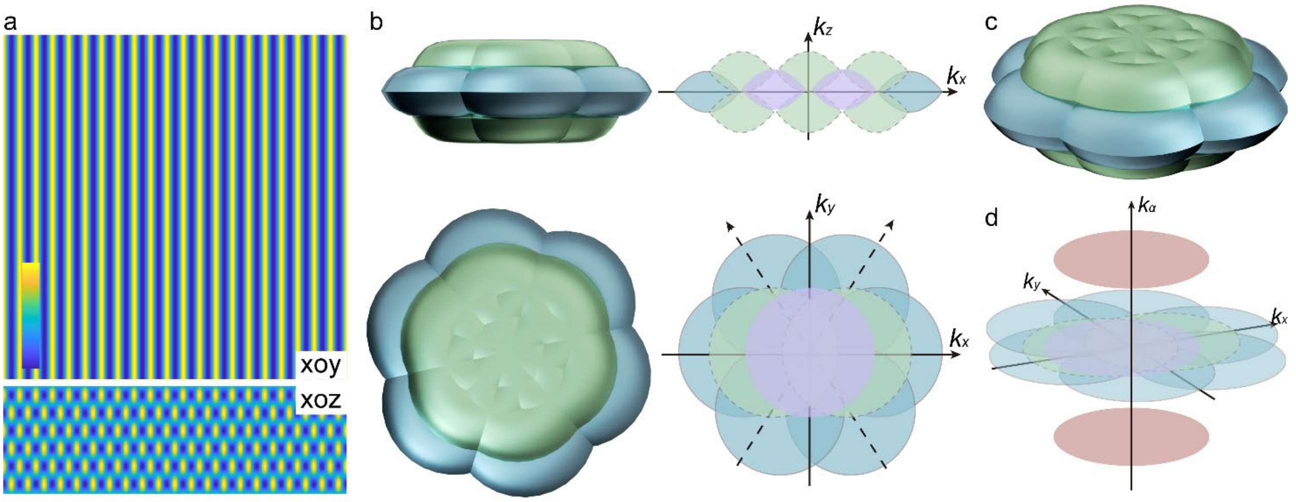

Supplementary Fig. 1 | Principle of 3DSIM. a, The intensity distribution in the *xoy* and *xoz* plane. **b**, Shifted 3D frequency domain fills the leaky cone of OTF and **c** doubles the spectrum range, with **d** the further expansion of polarization dimension (red color). Purple, green, blue, and red color represent 0th, $\pm$ 1st, and $\pm$ 2nd spectrum, and the polarization information, respectively. At colour bars in **a**, in the format "Instensity [.]".

Supplementary Note 1. Principle of Open-3DSIM

As shown in **Fig. SN1(a)**, 3DSIM presents an intensity pattern in the *xoy* and *xoz* plane at the objective back focal plane. So, the illumination pattern sequence $\boldsymbol{I}_{\theta,\varphi}(\boldsymbol{x},\boldsymbol{y},\boldsymbol{z})$ can be expressed as[1]:

$$\boldsymbol{I}_{\theta,\varphi}(\boldsymbol{x},\boldsymbol{y},\boldsymbol{z}) = I_0\left|1 + 2m \cdot e^{j2\pi p_z \boldsymbol{z}} \cdot cos\left[2\pi p_{xy} \cdot \boldsymbol{r} + \varphi\right]\right|^2 \tag{1}$$

Where I_0 denotes the intensity of illumination, m denotes the modulation depth of the focal plane. $p_{xy} = \frac{nsin\psi}{\lambda}$, $p_z = \frac{n(1-cos\psi)}{\lambda}$ are the spatial frequency of pattern in the *xoy* and *xoz* plane, and $\psi = \arcsin{(NA/n)}$ is the polar angle of incidence, NA is the numerical aperture, λ is the excitation wavelength, n is the refractive index of the medium inside the illuminated specimen, $\boldsymbol{r} = cos\theta\boldsymbol{x} + sin\theta\boldsymbol{y}$ is the pattern vector in the *xoy* plane, $\boldsymbol{x}$ and $\boldsymbol{y}$ are the unit vectors in *x* and *y* direction, θ and φ are the angle and phase of the illumination pattern respectively.

Expand the square term of $\boldsymbol{I}_{\theta,\varphi}(\boldsymbol{x},\boldsymbol{y},\boldsymbol{z})$, a simplified expression can be obtained:

$$\boldsymbol{I}_{\theta,\varphi}(\boldsymbol{r},\boldsymbol{z}) = I_0\left[1 + 2m^2 + 4m \cdot cos2\pi p_z\boldsymbol{z} \cdot cos\left(2\pi p_{x,y}\boldsymbol{r} + \varphi\right) + 2m^2 cos\left(4\pi p_{x,y}\boldsymbol{r} + 2\varphi\right)\right] \tag{2}$$

So, the illumination pattern can be decomposed into the superposition of zero, first, and second harmonics with the relative weight of $a_0 = 1 + 2m^2$, $a_{1z}(\boldsymbol{z}) = 4m \cdot cos2\pi p_z\boldsymbol{z}$ (let $a_1 = 4m$) and $a_2 = 2m^2$, where only $a_{1z}(\boldsymbol{z})$ is relative to *z*, and a_0, a_2 are constants only relative to the modulation depth.

When the illumination pattern sequence $\boldsymbol{I}_{\theta,\varphi}(\boldsymbol{r},\boldsymbol{z})$ excites the sample, the emission distribution of fluorescence $\boldsymbol{D}_{\theta,\varphi}(\boldsymbol{r},\boldsymbol{z})$ acquired by a CCD camera can be expressed as:

$$\boldsymbol{D}_{\theta,\varphi}(\boldsymbol{r},\boldsymbol{z}) = \left[\boldsymbol{S}(\boldsymbol{r},\boldsymbol{z}) \cdot \boldsymbol{I}_{\theta,\varphi}(\boldsymbol{r},\boldsymbol{z})\right] \otimes H(\boldsymbol{r},\boldsymbol{z}) \tag{3}$$

Where $\boldsymbol{S}(\boldsymbol{r},\boldsymbol{z})$ denotes the fluorophores' emission light distribution, $H(\boldsymbol{r},\boldsymbol{z})$ denotes the 3D point spread function. Further, its 3D frequency domain expression in the *xoy* plane can be given by:

$$\begin{aligned}
&\boldsymbol{D}_{\theta,\varphi}\left(k_{x,y},k_z\right) \\
&\quad= \left[\boldsymbol{S}\left(k_{x,y},k_z\right) \otimes \boldsymbol{I}_{\theta,\varphi}\left(k_{x,y},k_z\right)\right] \cdot H\left(k_{x,y},k_z\right) \\
&\quad= I_0\{a_0 \cdot \boldsymbol{S}\left(k_{x,y},k_z\right) + a_1(k_z) \cdot \left[\boldsymbol{S}\left(k_{x,y} - p_{x,y},k_z\right)e^{j\varphi} + \boldsymbol{S}\left(k_{x,y} + p_{x,y},k_z\right)e^{-j\varphi}\right] \\
&\qquad+ a_2\left[\boldsymbol{S}\left(k_{x,y} - 2 \cdot p_{x,y},k_z\right)e^{j2\varphi} + \boldsymbol{S}\left(k_{x,y} + 2 \cdot p_{x,y},k_z\right)e^{-j2\varphi}\right]\} \cdot H\left(k_{x,y},k_z\right)
\end{aligned} \tag{4}$$

Where the 1D frequency domain of $a_{1z}(\boldsymbol{z})$ can be expressed as $a_{1z}(\boldsymbol{k}) = a_1 \cdot [\delta(k - p_z) + \delta(k + p_z)]$, So the 3D frequency domain of $a_1(k_z) \cdot \boldsymbol{S}\left(k_{x,y} \pm p_{x,y}, k_z\right)$ can be expressed as $2m \cdot [\boldsymbol{S}(k_{x,y} \pm p_{x,y}, k_z - p_z) + \boldsymbol{S}(k_{x,y} \pm p_{x,y}, k_z + p_z)]$.

In each angle of the illumination pattern, five even phases should be used to resolve the $\boldsymbol{S}(k_{x,y}, k_z)$, $\boldsymbol{S}(k_{x,y} \pm p_{x,y}, k_z - p_z) + \boldsymbol{S}(k_{x,y} \pm p_{x,y}, k_z + p_z)$ and $\boldsymbol{S}(k_{x,y} \pm 2p_{x,y}, k_z)$. Therefore, five equations can be listed in the format of the matrix:

$$\begin{bmatrix} \boldsymbol{D}_{\theta,\varphi_1}(k) \\ \boldsymbol{D}_{\theta,\varphi_2}(k) \\ \boldsymbol{D}_{\theta,\varphi_3}(k) \\ \boldsymbol{D}_{\theta,\varphi_4}(k) \\ \boldsymbol{D}_{\theta,\varphi_5}(k) \end{bmatrix} = I_0 A \begin{bmatrix} \boldsymbol{S}(k_{x,y}, k_z) \cdot H(k_{x,y}, k_z) \\ [\boldsymbol{S}(k_{x,y} - p_{x,y}, k_z - p_z) + \boldsymbol{S}(k_{x,y} - p_{x,y}, k_z + p_z)] \cdot H(k_{x,y}, k_z) \\ [\boldsymbol{S}(k_{x,y} + p_{x,y}, k_z - p_z) + \boldsymbol{S}(k_{x,y} + p_{x,y}, k_z + p_z)] \cdot H(k_{x,y}, k_z) \\ \boldsymbol{S}(k_{x,y} - 2p_{x,y}, k_z) \cdot H(k_{x,y}, k_z) \\ \boldsymbol{S}(k_{x,y} + 2p_{x,y}, k_z) \cdot H(k_{x,y}, k_z) \end{bmatrix} \tag{5}$$

We make $A = \begin{bmatrix} a_0 & a_1 e^{j\varphi_1} & a_1 e^{-j\varphi_1} & a_2 e^{j2\varphi_1} & a_2 e^{-j2\varphi_1} \\ a_0 & a_1 e^{j\varphi_2} & a_1 e^{-j\varphi_2} & a_2 e^{j2\varphi_2} & a_2 e^{-j2\varphi_2} \\ a_0 & a_1 e^{j\varphi_3} & a_1 e^{-j\varphi_3} & a_2 e^{j2\varphi_3} & a_2 e^{-j2\varphi_3} \\ a_0 & a_1 e^{j\varphi_4} & a_1 e^{-j\varphi_4} & a_2 e^{j2\varphi_4} & a_2 e^{-j2\varphi_4} \\ a_0 & a_1 e^{j\varphi_5} & a_1 e^{-j\varphi_5} & a_2 e^{j2\varphi_5} & a_2 e^{-j2\varphi_5} \end{bmatrix}$, so the five frequency components $\boldsymbol{C}_{0,\pm1,\pm2}(k)$ can be given by:

$$\begin{bmatrix} \boldsymbol{C}_0(k) \\ \boldsymbol{C}_{-1}(k) \\ \boldsymbol{C}_{+1}(k) \\ \boldsymbol{C}_{-2}(k) \\ \boldsymbol{C}_{+2}(k) \end{bmatrix} = \begin{bmatrix} \boldsymbol{S}(k_{x,y}, k_z) \cdot H(k_{x,y}, k_z) \\ [\boldsymbol{S}(k_{x,y} - p_{x,y}, k_z - p_z) + \boldsymbol{S}(k_{x,y} - p_{x,y}, k_z + p_z)] \cdot H(k_{x,y}, k_z) \\ [\boldsymbol{S}(k_{x,y} + p_{x,y}, k_z - p_z) + \boldsymbol{S}(k_{x,y} + p_{x,y}, k_z + p_z)] \cdot H(k_{x,y}, k_z) \\ \boldsymbol{S}(k_{x,y} - 2p_{x,y}, k_z) \cdot H(k_{x,y}, k_z) \\ \boldsymbol{S}(k_{x,y} + 2p_{x,y}, k_z) \cdot H(k_{x,y}, k_z) \end{bmatrix} = \frac{1}{I_0} A^{-1} \begin{bmatrix} \boldsymbol{D}_{\theta,\varphi_0}(k) \\ \boldsymbol{D}_{\theta,\varphi_1}(k) \\ \boldsymbol{D}_{\theta,\varphi_2}(k) \\ \boldsymbol{D}_{\theta,\varphi_3}(k) \\ \boldsymbol{D}_{\theta,\varphi_4}(k) \end{bmatrix} \tag{6}$$

Where $[\cdot]^{-1}$ is the symbol of the matrix inverse transform. Then five frequency components $\boldsymbol{C}_n(k)$ are shifted to the correct position $\boldsymbol{C}_{ns}(k)$ laterally. $n = 0,\ \pm1, \pm2$ represents the index of five frequency componets.

$$\boldsymbol{C}_{ns}(k) = F\{F^{-1}[\boldsymbol{C}_n(k)] \cdot e^{-jn2\pi p_{x,y}}\} \tag{7}$$

Where $F^{-1}[\cdot]$ denotes 3D inverse Fourier transform. So, the frequency domain of the initial super-resolution image $\boldsymbol{C}_{SR\_0}(k)$ as shown in **Fig. SN1(b)** can be expressed as:

$$\boldsymbol{C}_{SR\_0}(k) = \sum_{n=-2}^{2} \boldsymbol{C}_{ns}(k) \tag{8}$$

In the reconstruction of SIM, there are several typical problems: (1) Traditional parameter estimation may be wrong under low SNR or low modulation depth[2, 3]; (2) The peak of high

frequency componets may result in honeycomb artifacts[2]; (3) The abnormal spectrum may cause sidelobe artifacts[4]; (4) The noise in high-frequency part may cause hammerstroke artifacts[2]; and (5) The traditional blur of high-frequency part will decrease the weak information in sample[5].

To sovle these problems, we firstly proposed an adaptive parameter estimation method as shown in **Fig. 1(a)**. This method can greatly improve the correctness of parameter estimation under low SNR using both 1st and 2nd frequency componets.

Then, to reduce the honeycomb artifacts (typical honeycomb artifacts can be seen in the OMX result in **Extended Data Fig. 2(b)**, the first spectrum optimization of Open-3DSIM is to use a notch-filter to suppress the high-frequency peaks.

$$\boldsymbol{C}_{SR\_1}(k) = \sum_{n=-2}^{2} \boldsymbol{C}_{ns}(k) \cdot notch\,(x,y,z,n) \cdot\ OTF^{att}(x,y,z,n) \qquad (9)$$

Where $notch(x,y,z,0) = 1 - d \cdot exp\left[\left(\frac{x^2+y^2}{|p_{x,y}|^2} + \frac{z^2}{|p_z|^2}\right)/2/w^2\right]$ is the notch-filter designed according to the estimated frequency vector on the *xoy* and *yoz* plane, $notch(x,y,z,n)$ is the corresponding shifted notchfilter in frequency-domain position on the base of $notch(x,y,z,0)$. $OTF(x,y,z,n)$ is the leaky-cone-shaped optical transfer function (OTF) shifted to the *n*-th place in the frequency domain, att is the frequency attenuation, and d and w are the notch depth and width respectively. To make the notch-filter applicable to samples with different layers and different wavelengths, the notch-filter is designed according to $p_{x,y}$ and p_z, which will greatly improve the universality and user-friendliness of Open-3DSIM with a fixed preset d and w.

However, as shown in **Extended Data Fig. 2(a)**, the combination of notched frequency components will still cause sidelobe artifacts because of the patchy features in the combined frequency domain. And the excessive notch will cause the loss of high-frequency signal, so we designed a spatial sum of $OTF \cdot notch$ as OTF_{notch} to design the filter for spectrum optimization.

$$OTF_{notch} = \sum_{n=-2}^{2} m(n) \cdot OTF(x,y,z,n) \cdot notch\,(x,y,z,n) \qquad (10)$$

Where $m(n)$ is the weight coefficient of different Fourier orders. It is noteworthy that the ideal spectrum of 3DSIM is smooth and even in the 3D domain. We think combined OTF (petal-shaped) is the ideal spectrum to be approached, and the directly combined notched spectrum is like the distribution of OTF_{notch}. Thus, we adopt the first filter called $Filter1(k) = \frac{Apo}{OTF_{notch}+w_1^2}$ to correct the abnormal frequency $\boldsymbol{C}_{SR\_1}(k)$ to $\boldsymbol{C}_{SR\_2}(k)$.

$$C_{SR\_2}(k) = C_{SR\_1}(k) \cdot \frac{Apo}{OTF_{notch}+w_1^2} \quad (11)$$

Where Apo is the apodization function in the 3D frequency domain, w_1 is the parameter to design $Filter1(k)$. As shown in **Extended Data Fig. 2(a)**, applying $Filter1(k)$ can greatly suppress the patchy features and high-frequency noise, so the ability to suppress artifacts is enhanced, but weak information is decreased because of the reduction in the edge of the petal-shaped spectrum. As a result, some weak information may be disappeared. What's more, with the involvement of w_1, the approach to the ideal spectrum and the compensation for the previous notch-filter is not complete. So, we adapt an extra filter called $Filter2(k) = \frac{Apo}{OTF_{notch}+w_2^2}$ to continuously approach the ideal spectrum and retain weak information of the reconstructed image. The final spectrum $C_{SR\_3}(k)$ can be expressed as:

$$C_{SR\_3}(k) = C_{SR\_2}(k) \cdot \frac{Apo}{OTF_{notch}+w_2^2} \quad (12)$$

The format of $Filter2(k)$ is the same as $Filter1(k)$, but with a relatively smaller w_2 (**Extended Data Fig. 3(a)(b)**), the proportion of high-frequency component can be increased. $Filter2(k)$ will greatly maintain the weak information. The cooperation of two filters of $C_{SR\_1}(k)$ and $C_{SR\_2}(k)$ can fill the spectrum hole caused by the previous notch operation and enhance the smoothness of the SIM spectrum. The proper selection of w_2 can greatly maintain the weak information with no observable hammerstroke artifacts as shown in **Supplementary Note 4**. And The effect of Filter1, Filter2, and the comparison of the spectrum between different algorithms can be seen in **Supplementary Note 3**.

So, the final super-resolution 3DSIM image I_{SR} can be finally expressed as:

$$I_{SR} = F^{-1}[C_{SR\_3}(k)] \quad (13)$$

Reference

1. Smith, C. et al. Structured illumination microscopy with noise-controlled image reconstructions. *Nat. Methods* **18**, 821-828 (2021).
2. Demmerle, J. et al. Strategic and practical guidelines for successful structured illumination microscopy. *Nat. Protoc.* **12**, 988-1010 (2017).
3. Karras, C. et al. Successful optimization of reconstruction parameters in structured illumination microscopy – a practical guide. *Opt. Commun.* **436**, 69–75 (2019).
4. Huang, X. et al. Fast, long-term, super-resolution imaging with Hessian structured illumination microscopy. *Nat. Biotechnol.* **36**, 451-459 (2018).

5. Wen, G. et al. High-fidelity structured illumination microscopy by point-spread-function engineering. *Light Sci. Appl.* **10**, 1-12 (2021).

Supplementary Note 2. Algorithm flow

The algorithm flow of Open-3DSIM is shown in **Extended Data Fig. 1**. Input is the raw data and physical parameters, and simulated or experimental OTF can be chosen. We can easily get wide-field images after summing all the images layer by layer. Then we calculate the module contrast (MCNR, used to evaluate the clarity of illumination pattern on different *z* layers) of every layer and use them as weights to average all the layers to a 2D image, then we estimate the pattern parameter (including frequency, angle, phase, and modulation depth) with the edge taper operation(can be chosen or not) on the raw image. Up-sampling was operated on the *xoy* Fourier plane to satisfy Nyquist sampling theory. The FFT distribution of phases 1-5 in a certain illumination angle has five peaks shown as the black arrow which represents the different frequency orders. The spectrum was then separated into five bands and shifted to the corresponding frequency place and the phase offset was corrected. Then a 3D notch-filter was conducted to suppress the high-frequency spike in the frequency domain and Filter1/Filter2 designed by the OTF was used to reduce noise and compensate for OTF blurring. After the inverse Fourier transform, reconstructed images can be obtained. In the end, polarization information was calculated by the raw image of different illumination angles, so the polarized 3D SIM was obtained.

Supplementary Note 3. The effect of spectrum optimization

In Open-3DSIM, we construct two-step spectrum optimization to reduce artifacts and improve the resolution. The notched image (fourier inverse transform of directly combined notched spectrum) and the image filtered after Filter1 and after Filter2 are listed at the top of **Extended Data Fig. 2(a)**, and the partially enlarged views of the *xoy* plane are listed on the center line. Note that the notched image has observable artifacts as the white arrow shows, but after two filters, the artifact has been greatly suppressed. And after Filter2, the resolution improved as the white profiles shown. The partially enlarged views of the *xoz* are shown at the bottom and can be seen that the resolution in the *xoz* plane has increased too. What's more, the frequency domain of the reconstructed images has been closer to the ideal petal-shaped uniform distribution with the use of filters.

What's more, we use the actin filament in **Fig. 2(a)** to compare the spectrum between different algorithms. It can be seen from **Extended Data Fig. 2(b)** that under the condition of an extremely low SNR, the high-frequency of the OMX spectrum is missing and the high-frequency spike is prominent, resulting in serious honeycomb artifacts. The high-frequency peak of SIMnoise is prominent, but the high-frequency center is sunk, resulting in the reduction of proportion between high and low frequency components, causing defocused backgrounds and abnormal spectrum. Through spectrum optimization, Open-3DSIM also maintains a uniform petal-shaped spectrum even under extremely low SNR, thus achieving good reconstruction results.

Supplementary Note 4. Guide for choosing parameters of spectrum optimization

In our work, we set all reconstructions with edegetaper (edge smoothing) and attenuation (*att* in eq(9)) equal to 0. To preserve edge and high-frequency information, we also recommend users keep the default settings. However, for the original image with lots of edge information, a reasonable set of some Edegetaper (typically 1-10 pixels for 512×512 images) can help to reduce the artifacts; In the case of serious high-frequency noise, a reasonable set of Attenuation between 0 and 0.5 will help to reduce the noise. Notchwidth1(w to construct OTF_{notch}) and Notchwidth2(w to construct notch-filter) are notch range parameters related to image size, and Notchdepth1(d to construct OTF_{notch}) and Notchdepth2(d to construct notch-filter) are constant notch depth. All reconstructions in this article maintain the default parameters of these four variables, and users are also recommended to keep the default parameters. However, in the process of spectrum analysis after reconstruction, if honeycomb artifacts caused by an insufficient notch (obvious high-frequency modulation point) are found, Notchdepth2 and Notchwidth2 can be appropriately increased, and Notchdepth1 and Notchwidth1 can be appropriately reduced.

To the users, we set w_1 and w_2 to help users to adjust parameters. As shown in **Extended Data Fig. 3(a)**, Filter1 generally presents a low-pass filter while Filter2 presents a high-pass filter. Larger w_1 will make Filter1 more obvious in suppressing the high-frequency information in the OTF of each spectrum, and smaller w_2 will enhance the high-frequency part of the petal-shaped spectrum (**Extended Data Fig. 4(b)**).

We also use the actin filament in **Extended Data Fig. 2** as an example, and the well-adjusted parameter is w_1 = 0.5 and w_2 = 0.1 in **Extended Data Fig. 3(c)**. Filter1 is mainly used for spectrum editing after abnormal reorganization in cooperation with the previous notch-filter. When w_1 is larger, the suppression of high-frequency noise at various spectral levels is more obvious, and the ability to suppress sidelobe artifacts is enhanced (**Extended Data Fig. 3(f)**). But the high-frequency information may be suppressed too. Filter2 is mainly used to preserve weak signals, but low w2 will amplify high-frequency noise and make the image too sharp with hammerstroke artifacts (**Extended Data Fig. 3(d, e)**). Users can adjust w_2 to achieve a balance between resolution and hammerstroke

artifacts. We suggest that for the image with a high SNR, we can appropriately reduce w_2 to magnify high-frequency information, and for the image with a low signal-to-noise ratio, we can appropriately increase w1 to suppress noise and artifact.

What's more, we list a table to list of parameters of our results as shown in **Supplementary Table 1**. In general, we set w_1 = 0.5 and w_2 = 0.1 to balance noise and artifacts and retain weak information. And the parameters usually do not need users to adjust, which brings great convenience.

Supplementary Note 5. Comparison between single-layer and multi-layer SIM reconstruction

To illustrate the necessity and motivation to develop the multi-layer SIM, we compare the reconstruction results of WF, HiFi-SIM, and Open-3DSIM as shown in **Extended Data Fig. 4(a, b)**. Although HiFi-SIM achieves a good effect of artifact removal and fidelity, it is still restricted to single-layer. When it comes to thick samples with serious defocused backgrounds, the reconstructed results of HiFi-SIM have more defocused backgrounds and defocused artifacts with no improvement in *xoz* resolution. By comparison, Open-3DSIM can better remove the defocus or artifacts, and improve the *xoz* resolution.

What's more, we did a quantitative evaluation of the *z*-axis resolution of multi-layer 3D SIM and single-layer 3DSIM. As shown in **Extended Data Fig. 4(c, d)**, taking the Argolight pentagon pattern and 100nm fluorescent beads of 488nm excitation wavelength as examples, we take 10 spatial points on Argolight and beads to make the intensity-pixel curve (processing with spline interpolation) on the z-axis as shown in **Extended Data Fig. 4(e, f)**. It can be seen that the half-height and full-width in the z-axis of WF, single-layer 3D SIM, and multi-layer 3D SIM are 1218nm, 1412nm, and 765nm for Argolight, respectively. And the half-height and full-width in the z-axis of WF, single-layer 3D SIM, and multi-layer 3D SIM are 596nm, 525nm, and 387nm for beads, respectively. At the same time, we use the PSFj plugin to quantitatively analyze the resolution of beads, and get that the resolution of the *xoy* plane and z-axis of WF is 251nm, 717nm, the resolution of the *xoy* plane and z-axis of single-layer 3DSIM is 120nm, 708nm, and the resolution of the *xoy* plane and z-axis of multi-layer 3DSIM is 118nm, 344nm. Therefore, from a quantitative point of view, we show that 3DSIM achieves double 3D resolution compared with WF and double *z*-resolution compared with single-layer 3DSIM.

We would like to stress that our comparison here to illustrate the advantages of multi-layer 3DSIM in improving *z*-axis resolution compared with single-layer 3DSIM. 2DSIM (or single-layer 3DSIM) has lower phototoxicity and faster imaging speed. On the contrary, multi-layer 3DSIM has the ability of whole-cell imaging with resolution improvement on z-axis. Users should reasonably select the required imaging method according to their needs.

Supplementary Note 6. Simulation on Open-3DSIM

We simulated the illumination process using an open-source 3D structure and resolution test image and reconstructed it using the Open3D-SIM algorithm after PSF blurring. The wide-field image, ground-truth image, and reconstructed image are shown in **Extended Data Fig. 5(a, b)**. It can be seen that the reconstructed image has improved resolution and removed the influence of defocused background with no observable artifact. And without post-processing methods such as RL deconvolution, the fidelity of Open-3DSIM is highly guaranteed.

Reference

1. Soubies, E. et al. Pocket Guide to Solve Inverse Problems with GlobalBioIm Inverse Problems. github 10.5281/zenodo.2624641 (2022).

Supplementary Note 7. Comparison of Weiner-based 3DSIM algorithms

To illustrate the performance of Open-3DSIM, we did a comparison between Open-3DSIM, AO-3DSIM, and the 3DSIM part of 4BSIM (simplified as 4BSIM) using their well-adjusted reconstructed results. It can be shown from **Extended Data Fig. 6(a, b)** that Open-3DSIM outperforms traditional Weiner-based Gustafsson-3DSIM algorithms including AO-3DSIM and 4BSIM with fewer artifacts and can retain weak information. It is noteworthy that, except that SIMnoise and Open-3DSIM did optimization on Weiner-based 3DSIM, other algorithms are completely based on traditional Cudasirecon (Gustafsson's 3DSIM) for certain hardware purposes. So, it is the reason that our manuscript mainly compares Open-3DSIM with SIMnoise and commercial OMX systems.

Supplementary Note 8. Comparison of various 3DSIM algorithms on Argolight

To prove the fidelity and performance of Open-3DSIM, we took photos of Algolight in the OMX system and compared the reconstruction performances of different algorithms in **Extended Data Fig. 7**. It can be seen that Open-3DSIM has an excellent performance in artifact and background removal compared with SIMnoise and OMX. In addition, Open-3DSIM resolution is significantly improved compared with SIMnoise due to the recovery of the high-frequency domain.

Supplementary Note 9. Comparison of various 3DSIM algorithms on various samples

Although SIMnoise obtains good reconstruction results by optimizing the wiener filter, Open-3DSIM transforms iterative optimization into parameter-based frequency domain optimization, which can better remove the effects of artifacts and further improve the resolution as shown in **Extended Data Fig. 8(a)**. We use image decorrelation method[1] to quanlify the resolution of different algorithms, finding that the resolutions in **Extended Data Fig. 8(a)** of SIMnoise and Open-3DSIM are 130.68nm and 111.24nm. But we also want to stess that the spectrum of Open-3DSIM is still limited in the petal-shaped spectrum without any deconvolution process to guarantee its high fidelity. The improvement of resolution is caused by the elimination of partial artifacts and background, as well as the reasonable proportion of redistribution between high-frequency and low-frequency spectrums.

And Open-3DSIM outperforms OMX-SIM in the respect of artifact removal and fidelity under low SNR in **Extended Data Fig. 8(b)**. We also test the three algorithms on the nuclear pore complex under high SNR. We find that after raising the contrast, we can still see the excellent artifact suppression ability and optical slicing effect of Open-3DSIM in **Extended Data Fig. 8(c)**.

Referrence

1. Descloux, A. Grußmayer, K. & Radenovic, A. Parameter-free image resolution estimation based on decorrelation analysis. *Nat. Methods* **16**(9): 918-924 (2019).

Supplementary Note 10. Multi-color imaging and reconstruction

We also reconstructed the three-color sample shown in **Extended Data Fig. 9**. Compared with the wide-field image, Open-3DSIM achieved double resolution in the *xyz* direction and achieved better optical slicing ability, which reflected the reconstruction ability of Open 3DSIM for multi-color samples.

Supplementary Note 11. Dipole orientation imaging

We also introduce the function of fluorescent dipole orientation imaging in Open3DSIM, so that users can obtain the polarization information directly and conveniently after reconstruction in **Extended Data Fig. 10(a)**. It is worth noting that structured light often has the problem of uneven light intensity in three directions, so it needs dense beads to correct the light intensity[1] as shown in **Extended Data Fig. 10(b)**. More samples of filament action in U2OS are shown in **Extended Data Fig. 10(c)** above, and the result shows accurate and correct dipole orientation imagings.

Reference

1. Zhanghao, K. et al. Super-resolution imaging of fluorescent dipoles via polarized structured illumination microscopy. *Nat. Commun.* **10**, 1-10 (2019).

Supplementary Table 1. Parameters and data origin used in our work.

Table SN1. Parameters and data origin used in our work.

Figure	Exwavelength(nm)	w_1	w_2	Data origin
Fig. 1(c)	488	0.5	0.1	OMX
Fig. 2(a)	568	0.5	0.1	OMX
Fig. 2(c)	488	0.5	0.1	SIMnoise
Fig. 2(d)	488	0.5	0.1	OMX
Fig. 2(e)	488, 561, 609	0.5,0.5,0.8	0.1,0.1,0.1	N-SIM
Fig. 2(f)	647	0.5	0.1	OMX
Fig. 2(g)	568	0.5	0.1	OMX
Fig. SN3(a, b)	488	0.5	0.1	fairSIM, OMX
Fig. SN4	488	0.8	0.1	fairSIM
Fig. SN5(a, b)	488	0.8	0.1	Simulation
Fig. SN6(a, b)	488,488	0.5,0.5	0.1,0.1	OMX, fairSIM
Fig. SN6(c, d)	488,488	0.5,0.5	0.1,0.1	OMX
Fig. SN7(a, b)	647,488	0.5,0.5	0.1,0.1	AO-3DSIM, 4BSIM
Fig. SN8(a, b)	488,488	0.5,0.5	0.1,0.1	OMX
Fig. SN9(a, b, c)	488,568,568	0.5,0.5,0.5	0.02,0.1,0.1	SIMnoise, OMX, OMX
Fig. SN10	405,488,568	0.5,0.5,0.5	0.1,0.1,0.1	OMX
Fig. SN11	488	0.5	0.1	OMX

Supplementary Table 2. List of the open-source data.

Table SN2. List of the open-source data.

File	Explanation	Exwavelength	Oil	Image size
OMX_Argolight_ex488_oil1518(253M).dv	OMX raw data	488nm	1.158	512×512×33
OMX_COS7_Nup_ex568_oil1514(192M).dv	OMX raw data	568nm	1.514	512×512×25
OMX_Mouse_section_ex568_oil1518(315M).dv	OMX raw data	568nm	1.518	512×512×41
OMX_U2OS_Actin_ex488_oil1518(77M).dv	OMX raw data	488nm	1.518	512×512×10
OMX_U2OS_Actin_ex568_oil1518(100M).dv	OMX raw data	568nm	1.512	512×512×13
SIMnoise_Beads_ex488_oil1512(63M).dv	SIMnoise raw data	488nm	1.512	512×512×33
SIMnoise_C127_Tubulin_ex488_oil1512(79M).dv	SIMnoise raw data	488nm	1.512	512×512×41
NSIM_U2OS_Actin_ex488_oil1512(83M).tif	NSIM raw data	488nm	1.512	1024×1024×11
Comparison	Reconstrcuted results and comaprisons	/	/	/
Install_Fiji_Screenshot.mp4	Guide vedio to install Fiji version	/	/	/
Parameter.zip	Paramters, OTFs	/	/	/
