# Supplementary material for: Open-3DSIM: an open-source three-dimensional structured illumination microscopy reconstruction platform
Source: Nat Methods. 2023 Jul 20;20(8):1183–6. doi: 10.1038/s41592-023-01958-0 (PMC10406603; doi:10.1038/s41592-023-01958-0)
Supplement: Supplementary file 2 — Reporting Summary [file 41592_2023_1958_MOESM2_ESM.pdf]

Corresponding author(s): PENG XI

Last updated by author(s): Mar 24, 2023

Reporting Summary

Nature Portfolio wishes to improve the reproducibility of the work that we publish. This form provides structure for consistency and transparency in reporting. For further information on Nature Portfolio policies, see our Editorial Policies and the Editorial Policy Checklist.

Statistics

For all statistical analyses, confirm that the following items are present in the figure legend, table legend, main text, or Methods section.

n/a	Confirmed	
☐	☒	The exact sample size (*n*) for each experimental group/condition, given as a discrete number and unit of measurement
☒	☐	A statement on whether measurements were taken from distinct samples or whether the same sample was measured repeatedly
☒	☐	The statistical test(s) used AND whether they are one- or two-sided<br>*Only common tests should be described solely by name; describe more complex techniques in the Methods section.*
☐	☒	A description of all covariates tested
☒	☐	A description of any assumptions or corrections, such as tests of normality and adjustment for multiple comparisons
☒	☐	A full description of the statistical parameters including central tendency (e.g. means) or other basic estimates (e.g. regression coefficient) AND variation (e.g. standard deviation) or associated estimates of uncertainty (e.g. confidence intervals)
☒	☐	For null hypothesis testing, the test statistic (e.g. *F*, *t*, *r*) with confidence intervals, effect sizes, degrees of freedom and *P* value noted<br>*Give P values as exact values whenever suitable.*
☒	☐	For Bayesian analysis, information on the choice of priors and Markov chain Monte Carlo settings
☒	☐	For hierarchical and complex designs, identification of the appropriate level for tests and full reporting of outcomes
☒	☐	Estimates of effect sizes (e.g. Cohen's *d*, Pearson's *r*), indicating how they were calculated

Our web collection on statistics for biologists contains articles on many of the points above.

Software and code

Policy information about availability of computer code

Data collection	We obtain data based on the commercial OMX-SIM system (DeltaVision OMX SR, GE, USA) using an oil immersion objective (Olympus, Japan, ×60 1.4 NA) and commercial N-SIM system (Nikon, Japan) using an oil immersion objective (CFI Apochromat, Japan, ×100 1.49 NA).<br>For the OMX system, 3D-SIM sequences were performed with a pixel size of 80nm and 125nm in the xoy and xoz plane (5 phases, 3 angles, and 15 raw images per plane). And for the N-SIM system, 3D-SIM sequences were performed with the pixel size of 65nm and 120nm in the xoy and xoz plane (5 phases, 3 angles, and 15 raw images per plane).<br>Samples in Fig. 2(c), S. Extended Data Fig. 8(a) are obtained from open-source data in SIMnoise (v1.0): https://data.4tu.nl/articles/_/12942932. Cos7 cell in Fig. 2(e) is obtained from Dr. Christophe Leterrier (Aix Marseille University) using the Nikon system. Samples in Extended Data Fig. 2(a), Extended Data Fig. 3(a), and Extended Data Fig. 4(b) are obtained from open-source data in fairSIM(v1.5.0): http://www.fairsim.org/. The simulated structure in Extended Data Fig. 5(a) is obtained from open-source 3D structure data(v1.2) at https://github.com/Biomedical-Imaging-Group/GlobalBioIm. The simulated resolution test image in Extended Data Fig. 5(b) is ISO12233:2000 (Imatest, American). Extended Data Fig. 6(a) is obtained from AO-3DSIM (v1.0.0)(https://www.ebi.ac.uk/biostudies/studies/S-BSST629). Extended Data Fig. 6(b) is obtained from 4BSIM (v1.0)16 (https://zenodo.org/record/6727773).
Data analysis	We use SIMnoise(v1.0), OMX-system, HiFi-SIM(v1.01), AO-3DSIM(v1.0.0) and 4BSIM(v1.0) for comparison. Image decorrelation(v1.1.8), PSFj(July 28, 2014), and Fiji is used to analyse the reconstruction results. The results of the statistics are generated by Imaris(v9.0.1), Visio(2016), Origin (2021b), and Adobe Illustrator(2020).

For manuscripts utilizing custom algorithms or software that are central to the research but not yet described in published literature, software must be made available to editors and reviewers. We strongly encourage code deposition in a community repository (e.g. GitHub). See the Nature Portfolio guidelines for submitting code & software for further information.

Data

Policy information about availability of data

All manuscripts must include a data availability statement. This statement should provide the following information, where applicable:

- Accession codes, unique identifiers, or web links for publicly available datasets
- A description of any restrictions on data availability
- For clinical datasets or third party data, please ensure that the statement adheres to our policy

The supplementary data, parameters, corresponding comparisons, and the install video of the ImageJ version have been uploaded on Figshare (https://figshare.com/articles/dataset/Open_3DSIM_DATA/21731315).

Human research participants

Policy information about studies involving human research participants and Sex and Gender in Research.

Reporting on sex and gender	This information has not been collected.
Population characteristics	N/A
Recruitment	N/A
Ethics oversight	N/A

Note that full information on the approval of the study protocol must also be provided in the manuscript.

Field-specific reporting

Please select the one below that is the best fit for your research. If you are not sure, read the appropriate sections before making your selection.

☒ Life sciences ☐ Behavioural & social sciences ☐ Ecological, evolutionary & environmental sciences

For a reference copy of the document with all sections, see nature.com/documents/nr-reporting-summary-flat.pdf

Life sciences study design

All studies must disclose on these points even when the disclosure is negative.

Sample size	In this work, we present a 3D-SIM reconstruction tool. No sample-size based statistics is involved.
Data exclusions	No data were excluded.
Replication	We have upload the software, data and parameters. Using those, users can obtain the only result at any computer. So, we confirm the reproducibility of the experimental findings.
Randomization	This is not relevant to my study. Because we our data is not large, so the data is not allocated into groups.
Blinding	This is not relevant to my study. Because our duplicated reconstruction results are similar.

Reporting for specific materials, systems and methods

We require information from authors about some types of materials, experimental systems and methods used in many studies. Here, indicate whether each material, system or method listed is relevant to your study. If you are not sure if a list item applies to your research, read the appropriate section before selecting a response.

Materials & experimental systems

n/a	Involved in the study	
☐	☒	Antibodies
☐	☒	Eukaryotic cell lines
☒	☐	Palaeontology and archaeology
☒	☐	Animals and other organisms
☒	☐	Clinical data
☒	☐	Dual use research of concern

Methods

n/a	Involved in the study	
☒	☐	ChIP-seq
☒	☐	Flow cytometry
☒	☐	MRI-based neuroimaging

Antibodies

Antibodies used	Alexa Flour 488, Alexa Fluor 555 and Alexa Fluor 568
Validation	Alexa Flour 488 Phalloidin (A12379, USA) was purchased from Invitrogen directly.<br>The mouse kidney sections with Alexa Fluor 568 labeled was purchased from ThermoFisher(F24630, American) directly.<br>The COS7 cells with Alexa Fluor 555 labeled were purchased from GATTA Quant(GATTA-Cells 1C) directly.

Eukaryotic cell lines

Policy information about cell lines and Sex and Gender in Research

Cell line source(s)	Human osteosarcoma U2-OS cell line(HTB-96) were purchased from ATCC.<br>The BPAE cells(F36924) were purchased from ThermoFisher.<br>The COS7 cells(GATTA-Cells 1C) were purchased from GATTA Quant.
Authentication	We directly purchased the cell line from ATCC/ThermoFisher/GATTA Quant which has been authenticated.
Mycoplasma contamination	We confirm that the cell line we used was tested negative for mycoplasma contamination.
Commonly misidentified lines<br>(See ICLAC register)	We don't use any commonly misidentified cell lines here.
