# Supplementary material for: Open-3DSIM: an open-source three-dimensional structured illumination microscopy reconstruction platform
Source: Nat Methods. 2023 Jul 20;20(8):1183–6. doi: 10.1038/s41592-023-01958-0 (PMC10406603; doi:10.1038/s41592-023-01958-0)
Supplement: Supplementary file 4 — This includes three platforms of Open-3DSIM. [file 41592_2023_1958_MOESM4_ESM.zip › Supplementary Software/Open_3DSIM_Exe_v2.1/User guide of Open-3DSIM (Exe_v2.0).pdf]

Software Introduction for Open-3DSIM (Exe)

Ruijie Cao

1. **System requirements**
   Our software is developed on Windows 10 / Windows 11 with MATLAB of R2018a (Mathworks^{R}).

2. **Environment needed**
   Our software needs MATLAB runtime (MATLAB runtime 2018a).

3. **Installation guide**
   The installation of Open-3DSIM (Matlab) follows the following steps:
   A. Install the MATLAB runtime 2018a (which can be downloaded from https://ww2.mathworks.cn/en/products/compiler/matlab-runtime.html).
   B. Open the exe application (from "Open_3DSIM_Exe_v2.0", which can be downloaded from the release(tags) in Github: https://github.com/Cao-ruijie/Open3DSIM).
   C. Then, you can run Open_3DSIM.m for the demo.

4. **Instruction**
   The following steps should be performed for Open_3DSIM reconstruction:
   A. Run Open3DSIM.exe, and the GUI will show as Fig. 1.
   B. Choose the format of raw data and click Set in ①. The file type should be *.tif, *.tiff, *.dv, or *.nd2, which can be derived from OMX (sequence: phase-depth-channel-time-angle), N-SIM (sequence: image (angle in rows and phase in columns)-depth-channel-time) or home-built 3D-SIM system image (sequence: phase-angle-depth-channel-time). Then click ①.
   C. Input the raw 3DSIM images in the "Input Image" folder. And click ② to choose the input file.
   D. Choose the output folder to restore results of the wide-field image (WF.tif), 3DSIM image (Open_3DSIM.tif), polarized SIM image (pSIM.tif), and polarization direction (cm.tif) as shown in ③.
   E. Then you should fill in all the parameters as shown in the image to continue the process. And click the button "Read" to read the image file as shown in ④.
   F. Click the button "Process" to process the image and conduct reconstruction as shown in ⑤. Simulated OTF or experimental OTF can be chosen.
   G. Fill the parameter λ_1 and λ_2 to click "Filter" button as shown in ⑥. Users can perform the filtering operations several times to obtain the best reconstruction, but in our test, the default parameters can obtain satisfactory results generally.
   H. If you want to get the polarized distribution of the fluorophore, please click the button "pSIM" as shown in ⑨.
   I. For more precise pSIM resolution, we provided the button "Calib" to calibrate the illumination intensity nonuniformity. Please click ⑦ and ⑧ to choose the calibration tif/tiff file. It is worth noting that light intensity calibration is important for solving polarization information, but this step can be ignored.
   J. Then you can get the results in the output directory.

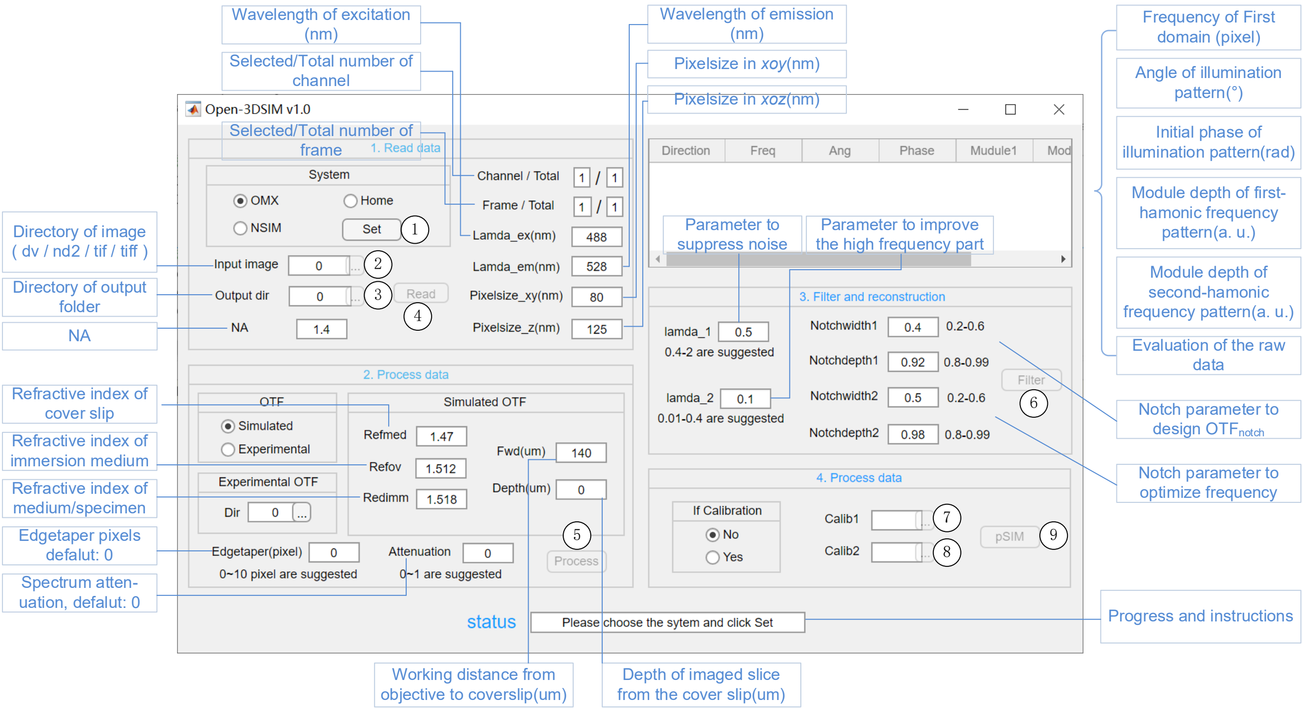

Fig. 1 GUI of Open-3DSIM (Exe version).

5. More data to test

More samples (raw data, parameters and comparisons) can be downloaded from Figshare (https://figshare.com/articles/dataset/Open_3DSIM_DATA/21731315).

6. Copyright

This code is programmed by Ruijie Cao and Prof. Peng Xi in Peking University. We claim an Apache liscence for Open-3DSIM.

References

A. Smith, C.S., Slotman, J.A., Schermelleh, L. *et al*. Structured illumination microscopy with noise-controlled image reconstructions. *Nat Methods* **18**, 821–828 (2021). https://doi.org/10.1038/s41592-021-01167-7

B. Wen, G., Li, S., Wang, L. *et al*. High-fidelity structured illumination microscopy by point-spread-function engineering. *Light Sci Appl* **10**, 70 (2021). https://doi.org/10.1038/s41377-021-00513-w

C. Zhanghao, K., Chen, X., Liu, W. *et al*. Super-resolution imaging of fluorescent dipoles via polarized structured illumination microscopy. *Nat Commun* **10**, 4694 (2019). https://doi.org/10.1038/s41467-019-12681-w

D. Besson S., Leigh R. *et al*. Bringing Open Data to Whole Slide Imaging. Digital Pathology ECDP 2019. Lecture Notes in Computer Science **11435**(2019). https://link.springer.com/chapter/10.1007/978-3-030-23937-4_1

E. Cris Luengo (2022). DIPimage (https://github.com/DIPlib/diplib), GitHub. Retrieved December 10, 2022.

If you have any questions, please contact caoruijie@stu.pku.edu.cn or xipeng@pku. edu.cn for help.
