# Supplementary material for: Open-3DSIM: an open-source three-dimensional structured illumination microscopy reconstruction platform
Source: Nat Methods. 2023 Jul 20;20(8):1183–6. doi: 10.1038/s41592-023-01958-0 (PMC10406603; doi:10.1038/s41592-023-01958-0)
Supplement: Supplementary file 4 — This includes three platforms of Open-3DSIM. [file 41592_2023_1958_MOESM4_ESM.zip › Supplementary Software/Open_3DSIM_Fiji_v2.1/User guide of Open-3DSIM (Fiji_v2.0).pdf]

User guide of Open-3DSIM (Fiji) v2.0

Ruijie Cao

1. System requirements

Our software is developed based on mixed programming of Java and MATLAB with Windows 10 / Windows 11.

2. Environment needed

Our software needs MATLAB runtime (MATLAB runtime 2018a) and Fiji.

3. Installation guide

The installation of Open-3DSIM (Fiji) takes the following steps:

A. Install the MATLAB runtime 2018a (which can be downloaded from https://ww2.mathworks.cn/en/products/compiler/matlab-runtime.html).

B. Install Fiji (which can be downloaded from https://imagej.net/software/fiji/downloads).

C. Open the source file "Open_3DSIM_Fiji_v2.0" (which can be downloaded from the release(tags) in Github : https://github.com/Cao-ruijie/Open3DSIM).

D. Put the five *.jar files (FILTER.jar, javabuilder.jar, PROCESS_DATA.jar, pSIM.jar, and READ_DATA.jar) under the "jars" folder of the directory of Fiji (typically .\Fiji.app\jars\), as shown in Fig 1(a).

E. Put the plugin (Open3DSIM-0.1.0-SNAPSHOT.jar) under the "plugins" folder of the directory of Fiji (typically .\Fiji.app\plugins\), as shown in Fig 1(c).

F. Then you can run Open_3DSIM for the demo in "Fiji>plugins>Open-3DSIM", as shown in Fig 1(d).

Note: We have uploaded a video to guide users to install and use Fiji version on Figshare (https://figshare.com/articles/dataset/Open_3DSIM_DATA/21731315), namely "Install_Fiji_Screenshot.mp4".

4. Instruction

The following steps should be performed for Open_3DSIM reconstruction:

A. Run Open3DSIM.exe, and the GUI will show as Fig. 2.

B. Input the raw 3DSIM images in the "Input Image" folder. This can be done by click the … button as ①. The file type should be *.tif, *.tiff, *.dv, or *.nd2. Then click ①.

C. Choose the input format, which can be derived from OMX (sequence: phase-depth-channel-time-angle), N-SIM (sequence: image (angle in rows and phase in columns)-depth-channel-time) or home-built 3D-SIM system image (sequence: phase-angle-depth-channel-time). Then you should fill in all the parameters as shown in the image to continue the process.

D. Click the button "Read" to read the image file as shown in ③ to read the input.

E. Click the button "Process" to process the image and conduct reconstruction as shown in ④. The simulated OTF or experimental OTF can be chosen.

F. Fill the parameter λ_1 and λ_2 to click "Filter" button as shown in ⑤. Users can perform the filtering operations several times to obtain the best reconstruction, but in our test, the default parameters can obtain satisfactory results generally.

G. If you want to get the polarized distribution of the fluorophore, please click the button “pSIM” as shown in ⑨.

H. For more precise pSIM resolution, we provided the button “Calib” to calibrate the intensity nonuniformity. Please click ⑥ and click ⑦ and ⑧ to choose the calibration *.tif/*.tiff file. It is worth noting that light intensity calibration is important for solving polarization information, but this step can be ignored.

I. Then you can get the results in the Fiji and on the same directory of input images.

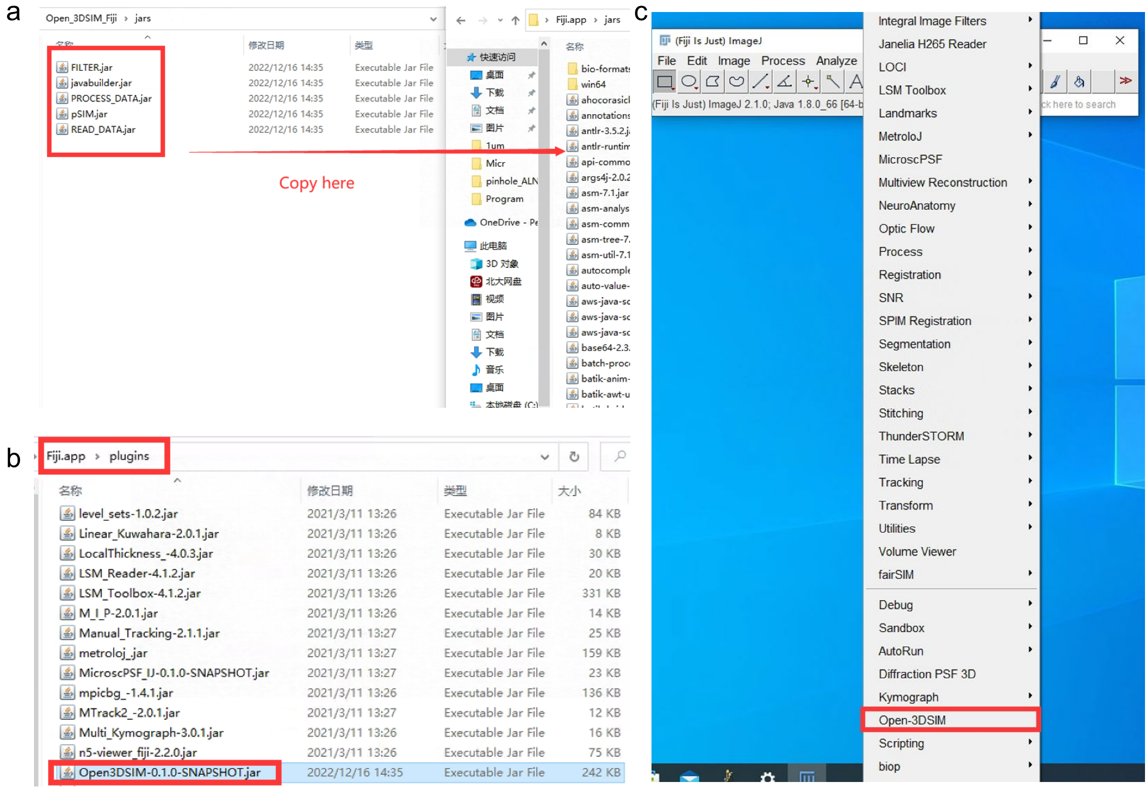

Fig. 1 Install of Open-3DSIM (Fiji).

5. More data to test

More samples (raw data, parameters and comparisons) can be downloaded from Figshare (https://figshare.com/articles/dataset/Open_3DSIM_DATA/21731315).

6. Copyright

This code is finished by finished by Ruijie Cao and Prof. Peng Xi in Peking University. We claim an Apache license for Open-3DSIM.

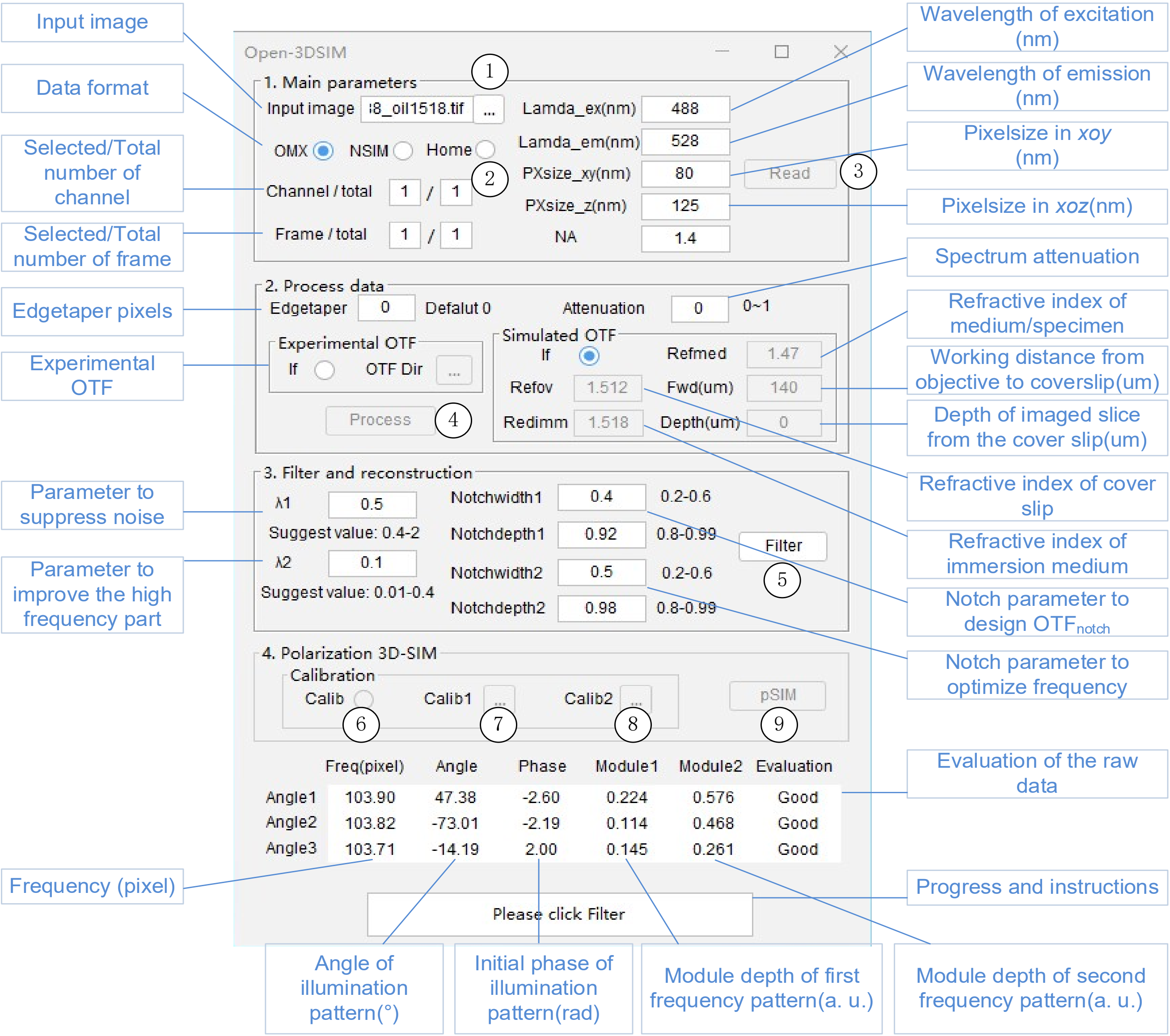

Fig. 2 GUI of Open-3DSIM (Fiji).

References

A. Smith, C.S., Slotman, J.A., Schermelleh, L. *et al*. Structured illumination microscopy with noise-controlled image reconstructions. *Nat Methods* **18**, 821–828 (2021). https://doi.org/10.1038/s41592-021-01167-7

B. Wen, G., Li, S., Wang, L. *et al*. High-fidelity structured illumination microscopy by point-spread-function engineering. *Light Sci Appl* **10**, 70 (2021). https://doi.org/10.1038/s41377-021-00513-w

C. Zhanghao, K., Chen, X., Liu, W. *et al*. Super-resolution imaging of fluorescent dipoles via polarized structured illumination microscopy. *Nat Commun* **10**, 4694 (2019). https://doi.org/10.1038/s41467-019-12681-w

D. Besson S., Leigh R. *et al*. Bringing Open Data to Whole Slide Imaging. Digital Pathology ECDP. Lecture Notes in Computer Science **11435**(2019). https://link.springer.com/chapter/10.1007/978-3-030-23937-4_1

E. Cris Luengo (2022). DIPimage (https://github.com/DIPlib/diplib), GitHub. Retrieved December 10, 2022.

If you have any questions, please contact caoruijie@stu.pku.edu.cn or xipeng@pku. edu.cn .
