# Supplementary material for: Open-3DSIM: an open-source three-dimensional structured illumination microscopy reconstruction platform
Source: Nat Methods. 2023 Jul 20;20(8):1183–6. doi: 10.1038/s41592-023-01958-0 (PMC10406603; doi:10.1038/s41592-023-01958-0)
Supplement: Supplementary file 4 — This includes three platforms of Open-3DSIM. [file 41592_2023_1958_MOESM4_ESM.zip › Supplementary Software/Open_3DSIM_MATLAB_v2.1/User guide of Open-3DSIM (MATLAB_v2.0).pdf]

User guide of Open-3DSIM (MATLAB)

Ruijie Cao

1. **System requirements**
   Our software is developed on Windows 10 / Windows 11 with MATLAB (Mathworks^{R}). MATLAB of R2018a or a later version is preferred.

2. **Environment needed**
   Our software needs MATLAB with open-source lib of Bio-Formats and Dip_Image to read *.dv/*.nd2/*.tif/*.tiff files and shift the 3D frequency domain. The libs are put in the "lib" folder for the convenience of users and developers.

3. **Installation guide**
   The installation of Open-3DSIM (MATLAB) follows the next steps:
   A. Open the source file "Open_3DSIM_MATLAB_v3.0" ((which can be downloaded from the release(tags) in Github: https://github.com/Cao-ruijie/Open3DSIM).
   B. Put the "input" and "output" files (in "test data" file) under the working directory.
   C. Run Open_3DSIM.m for the demo

4. **Instruction**
   The following steps should be performed for Open_3DSIM reconstruction:
   A. Put the raw SIM images in the "input" folder. The name should follow the format as: *.tif, *.tiff, *.nd2, or *.dv, which can be derived from OMX SR, N-SIM or a home-built 3D-SIM system.
   B. Open Open_3DSIM.m in MATLAB Editor Panel and input the required parameters.
   C. If you want a more accurate result, please put the experimental OTF into the "input" directory and correct the corresponding codes as the notes list.
   D. If you want a polarized map, please conduct the calibration step and put them into "input" file. It is worth noting that light intensity calibration is very important for solving polarization information, and polarization information without light intensity calibration is only for reference.
   E. Run Open_3DSIM.m
   F. The wide-field image (WF.tif), 3DSIM image (Open_3DSIM.tif), polarized SIM image (pSIM.tif), and polarization direction (cm.tif) are automatly saved in the "output" folder.

5. **Copyright**
   This code is finished by Ruijie Cao and Prof. Peng Xi in Peking University. We claim an Apache license for Open-3DSIM.

6. **More data to test**
   More samples (raw data, parameters and comparisons) can be downloaded from Figshare (https://figshare.com/articles/dataset/Open_3DSIM_DATA/21731315).

References

A. Smith, C.S., Slotman, J.A., Schermelleh, L. *et al*. Structured illumination microscopy with noise-controlled image reconstructions. *Nat Methods* **18**, 821–828 (2021). https://doi.org/10.1038/s41592-021-01167-7

B. Wen, G., Li, S., Wang, L. *et al*. High-fidelity structured illumination microscopy by point-spread-function engineering. *Light Sci Appl* **10**, 70 (2021). https://doi.org/10.1038/s41377-021-00513-w

C. Zhanghao, K., Chen, X., Liu, W. *et al*. Super-resolution imaging of fluorescent dipoles via polarized structured illumination microscopy. *Nat Commun* **10**, 4694 (2019). https://doi.org/10.1038/s41467-019-12681-w

D. Besson S., Leigh R. *et al*. Bringing Open Data to Whole Slide Imaging. Digital Pathology ECDP 2019. Lecture Notes in Computer Science **11435**(2019). https://link.springer.com/chapter/10.1007/978-3-030-23937-4_1

E. Cris Luengo (2022). DIPimage (https://github.com/DIPlib/diplib), GitHub. Retrieved December 10, 2022.

If you have any questions, please contact caoruijie@stu.pku.edu.cn or xipeng@pku. edu.cn for help.
